# Elevated cardiac troponin T indicating myocardial infarction or myocarditis–urgent cardiac catheter examination–or do we have to consider other reasons?

**DOI:** 10.1007/s12471-015-0694-5

**Published:** 2015-04-21

**Authors:** G. Ende, S. Richter, M.K. Schuler, S. Quick, R.H. Strasser, U. Speiser

**Affiliations:** 1 Herzzentrum Dresden, Universitätsklinik, Klinik für Innere Medizin und Kardiologie, Technische Universität Dresden, Fetscherstr. 76, 01307, Dresden, Deutschland; 2 Universitätsklinik, Medizinische Klinik 1, University Cancer Center, Technische Universität Dresden, Dresden, Deutschland

**Keywords:** Sarcoma, Troponin I, CMR, Magnetic resonance

## Abstract

Alveolar rhabdomyosarcoma is an aggressive tumour in adulthood, in which cardiac troponin T seems to be a tumour marker and course parameter. We present the clinical course of a young man suffering from this rare disease and the development of troponin T during therapy. Noninvasive cardiac imaging was used to exclude cardiac involvement, myocardial infarction or inflammation processes.

A 31-year-old man suffering from increasing chest pain radiating to the left shoulder and arm for 5 days was referred to our institution for further cardiological investigations. Only diabetes mellitus without any specific therapy was known as a potential cardiac risk factor.

A recurrence of a metastatic alveolar rhabdomyosarcoma with origin in the left sinus maxillaris was known in his medical history (Fig. 1a–c). The cancer was treated with neoadjuvant chemotherapy, followed by surgical resection and radiation.

**Fig. 1:**
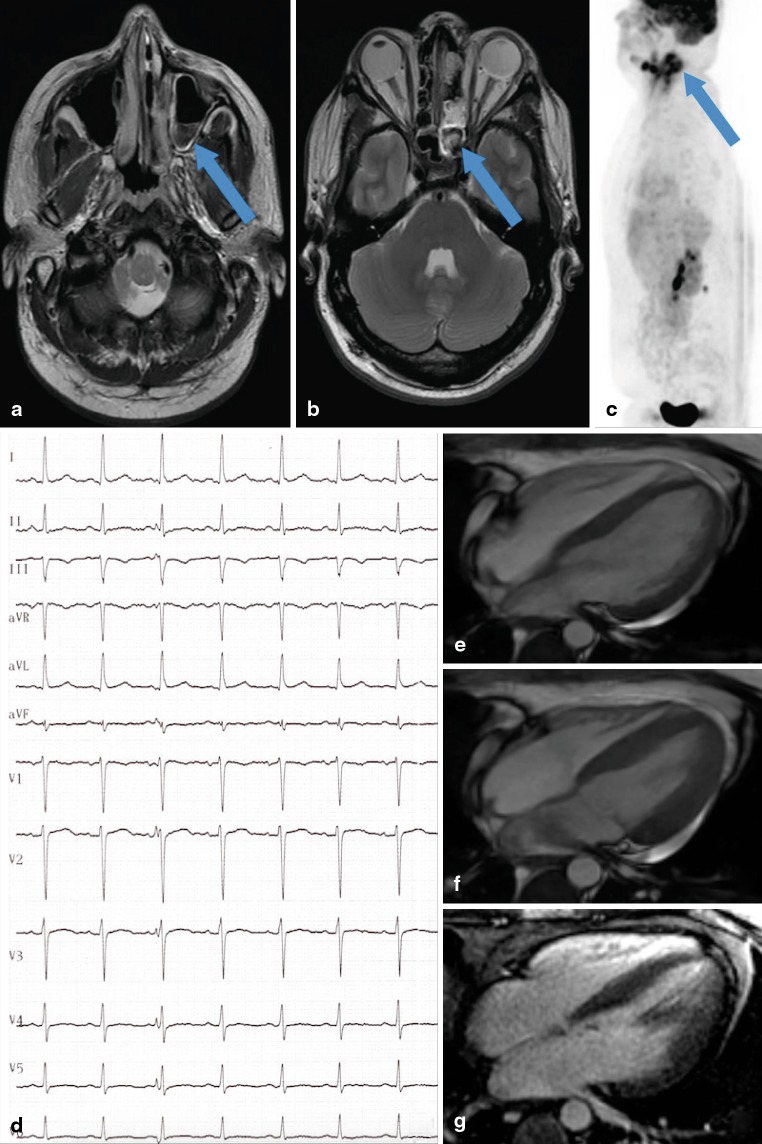
**a** Cranial magnetic resonance imaging (cMRI) of the head in T2 weighted sequences (*arrow* indicating rhabdomyosarcoma). **b** cMRI in T1 weighted sequences (*arrow* indicating rhabdomyosarcoma). **c** Positron emission tomography in lateral view (*arrow* indicating rhabdomyosarcoma). **d** Electrocardiogram with normal findings, no signs of acute myocardial infarction. **e** 3T cardiac magnetic resonance (CMR), 4 chamber view in diastole in FIESTA sequences. **f** 3T CMR, 4 chamber view in systole in FIESTA sequences. **g** 3T CMR, 4 chamber view in late gadolinium enhancement sequences without myocardial late enhancement

Electrocardiogram demonstrated normal sinus rhythm without evidence of acute myocardial ischaemia (Fig. 1d). Laboratory examination revealed a constellation of non-ST-segment myocardial infarction with cardiac troponin T (cTnT) 2146 ng/l (reference interval < 14 ng/l), serum creatine kinase (CK) 10.69 µmol/l (reference range < 3.17 µmol/l) and serum creatine kinase myocardial band (CK-MB) 2.59 µmol/l (reference range < 0.41 µmol/l). Transthoracic echocardiography displayed normal biventricular function and normal valve function; no wall motion disturbances or hints of right ventricular stress were found. For further differential diagnosis, especially for distinguishing myocardial ischaemia, myocarditis or myocardial damage in the context of the underlying disease, cardiac magnetic resonance (CMR) imaging was performed on the day of admission. Normal left and right ventricular function was confirmed in cine sequences (Fig. 1e–f, diastole and systole in the 4-chamber view). Black-blood T1 and black-blood T2 weighted sequences did not show any aspects of early enhancement or oedema. Late gadolinium enhancement images (Fig. 1g) excluded myocardial necrosis and fibrosis. CMR criteria for myocarditis were not fulfilled and, additionally, signs of myocardial infarction were not found. Therefore, after interdisciplinary discussion, the decision was made to prefer a noninvasive approach and not to send the patient for coronary angiography. Immediate chemotherapy with carboplatin and etoposide was initiated. During this therapy, his clinical symptoms continued to improve. The cardiac enzymes decreased significantly (cTnT 608 ng/l, CK 1.55 µmol/l, CK-MB 1.01 µmol/l on day 6 of therapy). In chemotherapy-free intervals, only the cTnT was slightly elevated without any changes in CK or CK-MB levels. In consideration of the diagnostic facts, the cause of the chest pain was the skin metastases in the areas of the left neck and shoulder.

Alveolar rhabdomyosarcoma presents a rare and aggressive tumour in adulthood. Cardiac troponin T seems to be usable as a tumour activity marker or a course parameter. This case highlights the differentiated consideration of the patient’s medical history and possibility of noninvasive imaging as the preferred approach instead of urgent invasive diagnostics, despite a typical laboratory constellation of myocardial infarction or myocarditis.

## Funding

None.

## Conflict of interests

None declared.

